# Independent Predictors of Cardiac Mortality and Hospitalization for Heart Failure in a Multi-Ethnic Asian ST-segment Elevation Myocardial Infarction Population Treated by Primary Percutaneous Coronary Intervention

**DOI:** 10.1038/s41598-019-46486-0

**Published:** 2019-07-11

**Authors:** Heerajnarain Bulluck, Huili Zheng, Mark Y. Chan, Nicolas Foin, David C. Foo, Chee W. Lee, Soo T. Lim, Anders Sahlen, Huay C. Tan, Jack W. Tan, Khim L. Tong, Aaron S. Wong, Philip E. Wong, Khung K. Yeo, Ling L. Foo, Terrance S. Chua, Tian H. Koh, Derek J. Hausenloy

**Affiliations:** 1 The Hatter Cardiovascular Institute, Institute of Cardiovascular Science, University College London, London, United Kingdom; 2 Cardiovascular and Metabolic Disorders Program, Duke-National University of Singapore, Singapore, Singapore; 3 National Heart Research Institute Singapore, National Heart Centre Singapore, Singapore, Singapore; 4 National Registry of Disease Office, Health Promotion Board, Singapore, Singapore; 5 National University Heart Centre, Singapore, Singapore; 6 Department of Cardiology, National Heart Centre Singapore, Singapore, Singapore; 7 Tan Tock Seng Hospital, Singapore, Singapore; 8 Khoo Teck Puat Hospital, Singapore, Singapore; 9 Karolinska Institutet, Department of Cardiology, Stockholm, Sweden; 10 Changi General Hospital, Singapore, Singapore; 11 Yong Loo Lin School of Medicine, National University Singapore, Singapore, Singapore; 12 Norfolk and Norwich University Hospital, Department of Cardiology, Norwich, UK; 13 The National Institute of Health Research University College London Hospitals Biomedical Research Centre, Research & Development, London, UK; 14 Tecnologico de Monterrey, Centro de Biotecnologia-FEMSA, Nuevo Leon, Mexico

**Keywords:** Interventional cardiology, Risk factors

## Abstract

We aimed to identify independent predictors of cardiac mortality and hospitalization for heart failure (HHF) from a real-world, multi-ethnic Asian registry [the Singapore Myocardial Infarction Registry] of ST-segment elevation myocardial infarction (STEMI) patients treated by primary percutaneous coronary intervention. 11,546 eligible STEMI patients between 2008 and 2015 were identified. In-hospital, 30-day and 1-year cardiac mortality and 1-year HHF rates were 6.4%, 6.8%, 8.3% and 5.2%, respectively. From the derivation cohort (70% of patients), age, Killip class and cardiac arrest, creatinine, hemoglobin and troponin on admission and left ventricular ejection fraction (LVEF) during hospitalization were predictors of in-hospital, 30-day and 1-year cardiac mortality. Previous ischemic heart disease (IHD) was a predictor of in-hospital and 30-day cardiac mortality only, whereas diabetes was a predictor of 1-year cardiac mortality only. Age, previous IHD and diabetes, Killip class, creatinine, hemoglobin and troponin on admission, symptom-to-balloon-time and LVEF were predictors of 1-year HHF. The c-statistics were 0.921, 0.901, 0.881, 0.869, respectively. Applying these models to the validation cohort (30% of patients) showed good fit and discrimination (c-statistic 0.922, 0.913, 0.903 and 0.855 respectively; misclassification rate 14.0%, 14.7%, 16.2% and 24.0% respectively). These predictors could be incorporated into specific risk scores to stratify reperfused STEMI patients by their risk level for targeted intervention.

## Introduction

Despite prompt reperfusion of ST-segment elevation myocardial infarction (STEMI) by primary percutaneous coronary intervention (PPCI), morbidity and mortality remain significant^1,2^. However, not all STEMI patients have the same prognosis. Those in the low-risk category have excellent prognosis^3^, whereas those with high-risk features have significantly worse outcomes with the risk of all-cause mortality reaching up to 35% at 30-day in those in the highest risk category^4^. Therefore early risk-stratification of STEMI patients is important to guide in-patient management and follow-up in order to improve clinical outcomes.

There are several risk scores available which can be used to risk-stratify STEMI patients at the time of hospitalization, and guide management^5^. The most extensively investigated, and validated risk scores are the Global Registry of Acute Coronary Events (GRACE)^6,7^, and the Thrombolysis in Myocardial Infarction (TIMI)^4^ risk scores, with the GRACE score performing better than the TIMI risk score in a recent meta-analysis^8^. The GRACE score can be used for all types of acute coronary syndromes (ACS), and can predict in-hospital and 6-month mortality. On the other hand, there is a specific TIMI risk score for STEMI^4^ to predict 30-day mortality. However, the derivation and validation cohorts for these risk scores consisted predominantly of non-Asian patients treated by thrombolysis.

About 60% of the world population resides in the Asia-Pacific region^1^ and Asians are known to have a different and higher cardiovascular risk profile than the western population^9^. However, there has been limited research conducted in the ACS population in this region so far due to factors such as language and cultural barriers, potential ethical issues, and differences in regulatory processes in individual Asian countries^10^, but efforts are under way with the establishment of the Asia-Pacific Real world evIdenCe on Outcome and Treatment of ACS (APRICOT) project^1^.

Singapore is a multi-ethnic country with Chinese (≈74%), Malays (≈13%) and Indians (≈9%) accounting for the majority of the population. It has a state-funded, mandatory, acute myocardial infarction (AMI) registry, called the Singapore Myocardial Infarction Registry (SMIR)^11^, with a dedicated team to collect data from all hospitals. We used this unbiased, real-world registry to determine independent variables on admission that would predict cardiac mortality during hospitalization, at 30-day and 1-year and 1-year hospitalization for heart failure (HHF) in patients presenting with a STEMI and reperfused by PPCI. These variables would subsequently be incorporated in risk scores to stratify reperfused STEMI patients by their risk level for targeted intervention early during hospitalization.

## Results

### Study population and baseline characteristics

A total of 11,546 STEMI patients reperfused by PPCI from 2008 to 2015 were identified in the SMIR. The overall cardiac mortality rate during hospitalization was 6.4% (741/11,546) and was 6.8% (780/11,546) at 30 days and 8.3% (956/11,546) at 1 year. Data on 1-year HHF was available from 2008 to 2013 only (n = 7,446). The 1-year HHF rate was 5.4% (399/7,446).

There were 8,082 reperfused STEMI patients in the derivation cohort. The median age was 57.6 (50.7–66.1) years old and 85.2% were male. The majority were Chinese (61.4%), followed by Malays (20.1%) and Indians (16.8%). Around a third of the cohort had a history of diabetes (28.1%) and half had a history of hypertension (52.2%), dyslipidemia (46.0%) and were current smokers (49.1%). 4.0% suffered a cardiac arrest in the ambulance or on admission to the emergency department and 8.7% were in cardiogenic shock at presentation. Half of the patients presented with an anterior STEMI (49.8%), and half had an abnormal initial troponin T or I (48.7%). The median symptom-to-balloon (S2B) time was 183 (120–310) minutes and the door-to-balloon (D2B) time was 65 (50–88) minutes.

There were 3,464 reperfused STEMI patients in the validation cohort. There was no significant difference in baseline characteristics between the derivation cohort and the validation cohort as summarized in Table 1 except for body mass index.

**Table 1:** Baseline characteristics in the derivation and validation cohort.

	Derivation cohort (n = 8082)	Validation cohort (n = 3464)	P
Age in years, median (IQR)	57.6 (50.7–66.1)	57.9 (50.9–66.0)	0.536
Age group, n (%)			0.597
<40	331 (4.1)	148 (4.3)	
40–59	4369 (54.1)	1833 (52.9)	
60–69	1952 (24.2)	857 (24.7)	
70–79	1016 (12.6)	461 (13.3)	
> = 80	414 (5.1)	165 (4.8)	
Gender, n (%)			0.361
Male	6885 (85.2)	2928 (84.5)	
Female	1197 (14.8)	536 (15.5)	
Ethnicity, n (%)			0.072
Chinese	4960 (61.4)	2167 (62.6)	
Malay	1622 (20.1)	722 (20.8)	
Indian	1360 (16.8)	515 (14.9)	
Others	140 (1.7)	60 (1.7)	
History of diabetes, n (%)	2273 (28.1)	980 (28.3)	0.853
History of hypertension, n (%)	4220 (52.2)	1796 (51.9)	0.72
History of dyslipidemia, n (%)	3712 (46.0)	1588 (45.9)	0.902
History of IHD, n (%)	1212 (15.0)	532 (15.4)	0.63
Current smoker, n (%)	3926 (49.1)	1718 (50.1)	0.34
BMI in kg/m^2^, median (IQR)	24.6 (22.4–27.3)	24.4 (22.2–27.1)	0.034
BMI group, n (%)			0.041
< = 23	2255 (30.9)	1032 (32.9)	
>23	5041 (69.1)	2101 (67.1)	
Killip class on admission, n (%)			0.297
I	6703 (83.0)	2849 (82.3)	
II	385 (4.8)	158 (4.6)	
III	288 (3.6)	148 (4.3)	
IV	705 (8.7)	309 (8.9)	
Cardiac arrest in ambulance/on admission, n (%)	319 (4.0)	155 (4.5)	0.19
Anterior STEMI on admission, n (%)	4022 (49.8)	1736 (50.1)	0.73
Creatinine on admission in µmol/L, median (IQR)	90 (76–109)	90 (77–109)	0.718
Creatinine group, n (%)			0.615
<70	1177 (14.6)	519 (15.1)	
70–105	4622 (57.3)	1944 (56.5)	
106–140	1501 (18.6)	675 (19.6)	
141–176	364 (4.5)	152 (4.4)	
177–353	269 (3.3)	100 (2.9)	
> = 354	135 (1.7)	54 (1.6)	
Hemoglobin on admission in g/dL, median (IQR)	14.6 (13.5–15.7)	14.7 (13.4–15.7)	0.58
Hemoglobin group, n (%)			0.633
<10	185 (2.3)	87 (2.5)	
10–11	547 (6.8)	258 (7.5)	
12–13	2031 (25.2)	863 (25.1)	
14–16	3693 (45.8)	1563 (45.4)	
> = 17	1609 (20.0)	674 (19.6)	
Elevated first troponin T/I on admission, n (%)	3938 (48.7)	1649 (47.6)	0.269
Blood sugar within 72 h from STEMI onset in mmol/L, median (IQR)	8.5 (6.7–12.2)	8.4 (6.7–12.1)	0.287
Total cholesterol within 72 h from STEMI onset in mmol/L, median (IQR)	5.1 (4.3–6.0)	5.1 (4.3–6.0)	0.514
HDL cholesterol within 72 h from STEMI onset in mmol/L, median (IQR)	1.0 (0.9–1.2)	1.0 (0.9–1.2)	0.431
LDL cholesterol within 72 h from STEMI onset in mmol/L, median (IQR)	3.4 (2.6–4.1)	3.3 (2.6–4.1)	0.264
Triglyceride within 72 h from STEMI onset in mmol/L, median (IQR)	1.4 (1.0–2.0)	1.4 (1.0–2.0)	0.98
HbA1c on admission in %, median (IQR)	6.1 (5.7–7.6)	6.1 (5.7–7.7)	0.97
S2B time in minutes, median (IQR)	183 (120–310)	180 (119–298)	0.118
S2B time group, n (%)			0.205
< = 180	3738 (49.2)	1654 (50.5)	
>180	3865 (50.8)	1622 (49.5)	
D2B in minutes, median (IQR)	65 (50–88)	64 (49–87)	0.435
Lowest LVEF during hospitalization in %, median (IQR)	45 (35–55)	45 (35–55)	0.874
LVEF group, n (%)			0.372
<40	2244 (29.5)	963 (30.0)	
40–50	3153 (41.5)	1286 (40.1)	
>50	2201 (29.0)	960 (29.9)	
Aspirin given during hospitalization, n (%)	7710 (95.4)	3278 (94.6)	0.078
Beta blocker given during hospitalization, n (%)	6793 (84.1)	2854 (82.4)	0.027
Lipid lowering therapy/statin given during hospitalization, n (%)	7710 (95.4)	3282 (94.8)	0.134
ACEI/ARB given during hospitalization, n (%)	5932 (73.4)	2537 (73.2)	0.86
Other anti-platelet given during hospitalization, n (%)	7800 (96.5)	3327 (96.1)	0.22
Aspirin given at discharge among those discharged alive, n (%)	7323 (97.1)	3102 (97.0)	0.935
Beta blocker given at discharge among those discharged alive, n (%)	6592 (87.4)	2766 (86.5)	0.229
Lipid lowering therapy/statin given at discharge among those discharged alive, n (%)	7377 (97.8)	3141 (98.3)	0.115
ACEI/ARB given at discharge among those discharged alive, n (%)	5568 (73.8)	2390 (74.8)	0.299
Other anti-platelet given at discharge among those discharged alive, n (%)	7405 (98.1)	3150 (98.5)	0.163

IQR: interquartile range; IHD: ischemic heart disease; BMI: body mass index; STEMI: ST-segment elevation myocardial infarction; S2B: symptom-to-balloon; D2B: door-to-balloon; LVEF: left ventricular ejection fraction; ACE: angiotensin converting enzyme inhibitor; ARB: angiotensin receptor blocker.

### Predictors of in-hospital, 30-day and 1-year cardiac mortality

Variables included in the univariable logistic regression are summarized in the Table 2. Variables included in the multivariable logistic regression are summarized in Table 3. Age, Killip class, cardiac arrest, creatinine, hemoglobin and troponin on admission and left ventricular ejection fraction (LVEF) during hospitalization were predictors of cardiac mortality at all 3 time points (in-hospital, 30-day and 1 year). Previous ischemic heart disease (IHD) - defined as a history of previous myocardial infarction, PCI or coronary artery bypass graft surgery - was a predictor of in-hospital and 30-day cardiac mortality only, whereas diabetes was a predictor of 1-year cardiac mortality only.

**Table 2:** Univariable regression model for prediction of cardiac mortality based on the derivation cohort.

	In-hospital cardiac mortality		30-day cardiac mortality		1-year cardiac mortality	
	Odds Ratio (95%CI)	P	Odds Ratio (95%CI)	P	Odds Ratio (95%CI)	P
Age (years)		<0.001		<0.001		<0.001
<40	1.00 (reference)		1.00 (reference)		1.00 (reference)	
40–59	2.95 (1.08–8.00)		2.64 (1.08–6.47)		3.21 (1.31–7.85)	
60–69	5.21 (1.91–14.22)		4.27 (1.73–10.53)		5.86 (2.39–14.38)	
70–79	12.53 (4.60–34.14)		10.59 (4.30–26.08)		14.04 (5.72–34.45)	
> = 80	21.75 (7.89–59.95)		18.11 (7.26–45.15)		23.59 (9.50–58.60)	
Gender		<0.001		<0.001		<0.001
Male	1.00 (reference)		1.00 (reference)		1.00 (reference)	
Female	2.52 (2.05–3.09)		2.42 (1.98–2.96)		2.48 (2.07–2.98)	
Ethnicity		0.105		0.105		0.072
Chinese	1.00 (reference)		1.00 (reference)		1.00 (reference)	
Malay	1.01 (0.80–1.27)		1.05 (0.84–1.31)		1.09 (0.90–1.33)	
Indian	0.77 (0.59–1.01)		0.77 (0.59–1.01)		0.77 (0.61–0.98)	
Others	1.52 (0.85–2.73)		1.44 (0.80–2.57)		1.23 (0.70–2.15)	
History of diabetes		<0.001		<0.001		<0.001
No	1.00 (reference)		1.00 (reference)		1.00 (reference)	
Yes	2.10 (1.74–2.52)		1.98 (1.66–2.37)		2.18 (1.86–2.57)	
History of hypertension		<0.001		<0.001		<0.001
No	1.00 (reference)		1.00 (reference)		1.00 (reference)	
Yes	1.75 (1.45–2.12)		1.75 (1.45–2.10)		2.01 (1.70–2.38)	
History of dyslipidemia		0.168		0.316		0.002
No	1.00 (reference)		1.00 (reference)		1.00 (reference)	
Yes	1.14 (0.95–1.36)		1.09 (0.92–1.31)		1.28 (1.09–1.50)	
History of IHD		0.014		0.011		<0.001
No	1.00 (reference)		1.00 (reference)		1.00 (reference)	
Yes	1.34 (1.06–1.70)		1.34 (1.07–1.69)		1.75 (1.44–2.12)	
Current smoker		<0.001		<0.001		<0.001
No	1.00 (reference)		1.00 (reference)		1.00 (reference)	
Yes	0.53 (0.43–0.64)		0.61 (0.50–0.74)		0.59 (0.49–0.70)	
BMI (kg/m^2^)		0.057		0.026		0.016
< = 23	1.00 (reference)		1.00 (reference)		1.00 (reference)	
>23	0.80 (0.63–1.01)		0.78 (0.62–0.97)		0.79 (0.65–0.96)	
Killip class on admission		<0.001		<0.001		<0.001
I	1.00 (reference)		1.00 (reference)		1.00 (reference)	
II	2.12 (1.36–3.31)		2.33 (1.55–3.51)		2.27 (1.59–3.25)	
III	7.88 (5.68–10.92)		7.66 (5.59–10.52)		7.52 (5.64–10.02)	
IV	15.24 (12.31–18.87)		13.58 (11.03–16.73)		11.58 (9.55–14.05)	
Cardiac arrest in ambulance/on admission		<0.001		<0.001		<0.001
No	1.00 (reference)		1.00 (reference)		1.00 (reference)	
Yes	12.87 (10.05–16.50)		11.07 (8.65–14.18)		9.37 (7.37–11.91)	
Anterior STEMI		0.001		<0.001		<0.001
No	1.00 (reference)		1.00 (reference)		1.00 (reference)	
Yes	1.37 (1.14–1.65)		1.44 (1.21–1.72)		1.37 (1.16–1.60)	
Creatinine on admission (µmol/L)		<0.001		<0.001		<0.001
<70	1.00 (reference)		1.00 (reference)		1.00 (reference)	
70–105	1.26 (0.83–1.92)		1.23 (0.83–1.82)		1.11 (0.80–1.55)	
106–140	4.83 (3.19–7.33)		4.57 (3.09–6.75)		3.82 (2.74–5.33)	
141–176	10.87 (6.87–17.20)		9.59 (6.19–14.87)		8.02 (5.47–11.76)	
177–353	17.71 (11.15–28.14)		15.10 (9.69–23.52)		13.08 (8.85–19.31)	
> = 354	9.68 (5.43–17.26)		7.99 (4.53–14.10)		9.50 (5.87–15.37)	
Hemoglobin on admission (g/dL)		<0.001		<0.001		<0.001
<10	1.00 (reference)		1.00 (reference)		1.00 (reference)	
11–11	0.86 (0.57–1.31)		0.84 (0.55–1.27)		0.78 (0.53–1.15)	
12–13	0.36 (0.24–0.53)		0.34 (0.23–0.50)		0.32 (0.23–0.46)	
14–16	0.16 (0.11–0.23)		0.16 (0.11–0.23)		0.14 (0.10–0.21)	
> = 17	0.14 (0.09–0.21)		0.16 (0.11–0.25)		0.14 (0.10–0.21)	
Elevated first troponin T/I on admission		<0.001		<0.001		<0.001
No	1.00 (reference)		1.00 (reference)		1.00 (reference)	
Yes	2.08 (1.72–2.52)		2.11 (1.75–2.54)		2.15 (1.82–2.54)	
S2B (minutes)		0.534		0.287		0.178
< = 180	1.00 (reference)		1.00 (reference)		1.00 (reference)	
>180	1.06 (0.88–1.29)		1.11 (0.92–1.34)		1.12 (0.95–1.33)	
Lowest LVEF during hospitalization (%)		<0.001		<0.001		<0.001
<40	1.00 (reference)		1.00 (reference)		1.00 (reference)	
40–50	0.12 (0.09–0.17)		0.14 (0.11–0.19)		0.15 (0.12–0.19)	
>50	0.10 (0.07–0.15)		0.10 (0.06–0.14)		0.12 (0.09–0.16)	

CI: confidence interval; IHD: ischemic heart disease; BMI: body mass index; STEMI: ST-segment elevation myocardial infarction; S2B: symptom-to-balloon; LVEF: left ventricular ejection fraction.

**Table 3:** Multivariable regression model for prediction of cardiac mortality based on the derivation cohort.

	In-hospital cardiac mortality		30-day cardiac mortality		1-year cardiac mortality	
	Odds Ratio (95%CI)	P	Odds Ratio (95%CI)	P	Odds Ratio (95%CI)	P
Age (years)		<0.001		<0.001		<0.001
<40	1.00 (reference)		1.00 (reference)		1.00 (reference)	
40–59	4.26 (0.58–31.41)		2.91 (0.70–12.16)		3.84 (0.93–15.93)	
60–69	5.92 (0.80–44.08)		3.42 (0.81–14.48)		5.55 (1.33–23.20)	
70–79	9.46 (1.26–70.99)		5.94 (1.39–25.39)		9.57 (2.27–40.33)	
> = 80	18.48 (2.44–140.17)		10.71 (2.47–46.46)		17.26 (4.04–73.63)	
Gender	Not significant		Not significant		Not significant	
Male						
Female						
History of diabetes	Not significant		Not significant			0.014
No					1.00 (reference)	
Yes					1.33 (1.06–1.67)	
History of hypertension	Not significant		Not significant		Not significant	
No						
Yes						
History of dyslipidemia	NA		NA		Not significant	
No						
Yes						
History of IHD		0.001		0.006	Not significant	
No	1.00 (reference)		1.00 (reference)			
Yes	0.55 (0.38–0.80)		0.62 (0.44–0.87)			
Current smoker	Not significant		Not significant		Not significant	
No						
Yes						
BMI (kg/m^2^) < = 23 >23	Not significant		Not significant		Not significant	
Killip class on admission		<0.001		<0.001		<0.001
I	1.00 (reference)		1.00 (reference)		1.00 (reference)	
II	1.07 (0.59–1.93)		1.20 (0.71–2.03)		1.13 (0.73–1.76)	
III	2.77 (1.79–4.31)		2.70 (1.79–4.09)		2.42 (1.68–3.48)	
IV	4.24 (3.11–5.77)		3.75 (2.79–5.05)		3.27 (2.50–4.27)	
Cardiac arrest in ambulance/on admission		<0.001		<0.001		<0.001
No	1.00 (reference)		1.00 (reference)		1.00 (reference)	
Yes	6.61 (4.52–9.65)		5.09 (3.52–7.37)		4.55 (3.20–6.46)	
Anterior STEMI	Not significant		Not significant		Not significant	
No						
Yes						
Creatinine on admission (µmol/L)		<0.001		<0.001		<0.001
<70	1.00 (reference)		1.00 (reference)		1.00 (reference)	
70–105	1.26 (0.73–2.16)		1.19 (0.73–1.95)		1.11 (0.75–1.64)	
106–140	2.36 (1.36–4.09)		2.29 (1.39–3.77)		1.92 (1.28–2.89)	
141–176	3.74 (2.01–6.96)		3.16 (1.77–5.62)		2.55 (1.57–4.13)	
177–353	5.04 (2.67–9.52)		4.08 (2.26–7.38)		3.25 (1.97–5.38)	
> = 354	4.37 (1.97–9.70)		2.94 (1.36–6.38)		3.79 (2.05–6.99)	
Hemoglobin on admission (g/dL)		<0.001		0.001		<0.001
<10	1.00 (reference)		1.00 (reference)		1.00 (reference)	
10–11	1.94 (1.05–3.59)		1.57 (0.88–2.80)		1.47 (0.87–2.46)	
12–13	1.13 (0.62–2.06)		0.87 (0.50–1.53)		0.96 (0.59–1.59)	
14–16	0.73 (0.39–1.35)		0.59 (0.33–1.05)		0.67 (0.40–1.12)	
> = 17	0.77 (0.38–1.56)		0.81 (0.43–1.54)		0.89 (0.50–1.57)	
Elevated first troponin T/I on admission		<0.001		<0.001		<0.001
No	1.00 (reference)		1.00 (reference)		1.00 (reference)	
Yes	1.69 (1.27–2.25)		1.76 (1.35–2.30)		1.68 (1.34–2.12)	
S2B (minutes) < = 180 >180	Not significant		Not significant		Not significant	
Lowest LVEF during hospitalization (%)		<0.001		<0.001		<0.001
<40	1.00 (reference)		1.00 (reference)		1.00 (reference)	
40–50	0.21 (0.15–0.30)		0.24 (0.18–0.33)		0.25 (0.19–0.32)	
>50	0.25 (0.16–0.38)		0.22 (0.14–0.34)		0.27 (0.19–0.37)	

CI: confidence interval; IHD: ischemic heart disease; BMI: body mass index; STEMI: ST-segment elevation myocardial infarction; S2B: symptom-to-balloon; LVEF: left ventricular ejection fraction; NA: not applicable.

**Figure 1:**
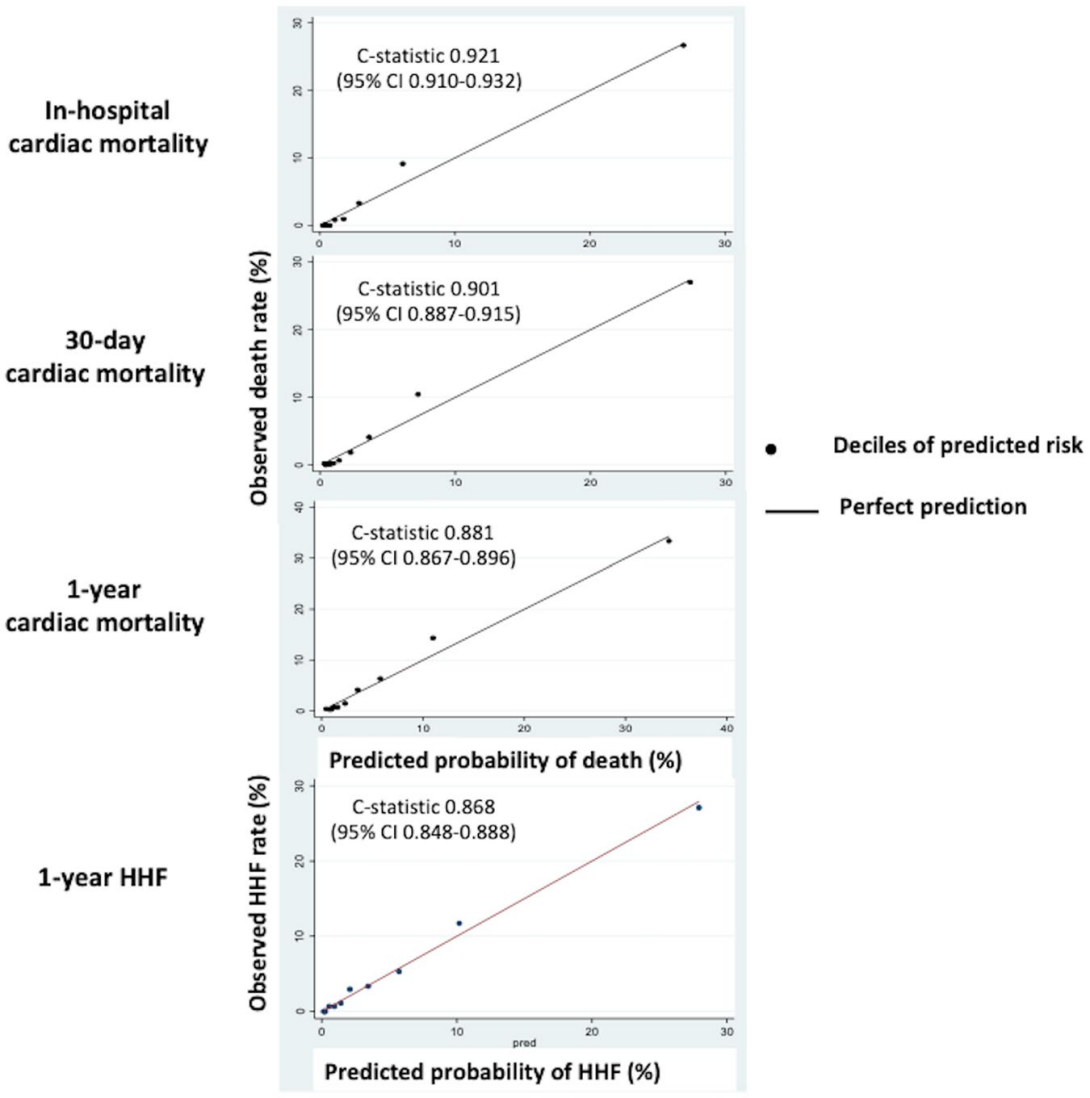
Internal validation of the models in the derivation cohort. There was good calibration of the models for in-hospital, 30-day and 1-year cardiac death and 1-year HHF with c-statistic of 0.921, 0.901, 0.881 and 0.868 respectively.

Calibration of the predictions from these models was assessed by comparing the average predictions to the actual cardiac mortality across deciles and was found to be excellent as shown in Fig. 1. Internal validation showed good fit of the models with a c-statistic of 0.921 (95%CI 0.910–0.932) for in-hospital cardiac mortality, 0.901 (95%CI 0.887–0.915) for 30-day cardiac mortality and 0.881 (95%CI 0.867–0.896) for 1-year cardiac mortality with bootstrapping techniques.

**Figure 2:**
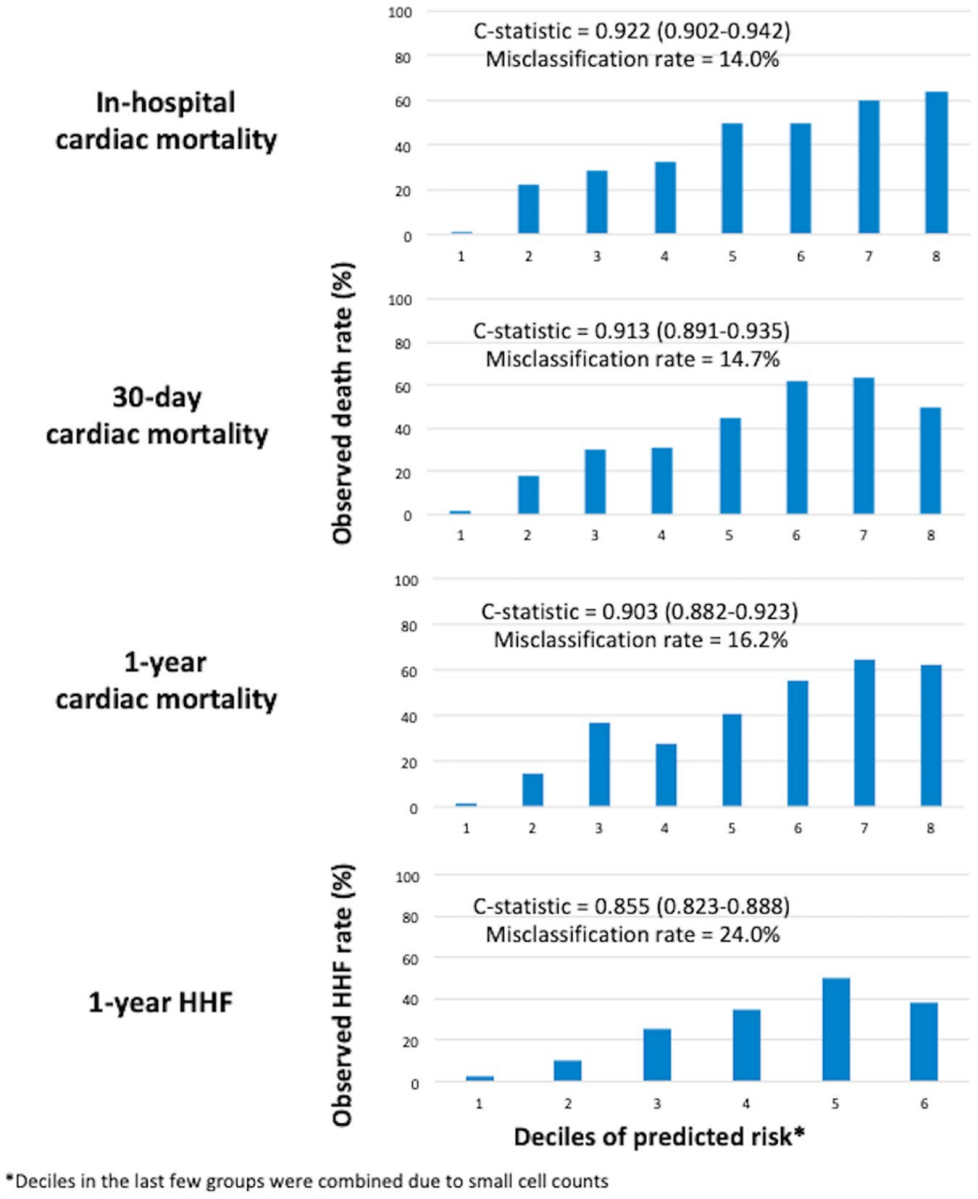
External validation of the models in the validation cohort. There was good discrimination of the models for in-hospital, 30-day and 1-year cardiac death and 1-year HHF with c-statistic of 0.922, 0.913, 0.903 and 0.855 respectively.

External validation was performed in the validation cohort of 3,464 patients. The model performed very well as shown in Fig. 2 with a c-statistic of 0.922 (95%CI 0.902–0.942) for in-hospital cardiac mortality, 0.913 (95%CI 0.891–0.935) for 30-day cardiac mortality and 0.903 (95%CI 0.882–0.923) for 1-year cardiac mortality. The misclassification rate for in-hospital, 30-day and 1-year cardiac mortality was 14.0%, 14.7% and 16.2% respectively.

### Predictors of 1-year hospitalization for heart failure

The derivation cohort consisted of 5,395 reperfused STEMI patients and the validation cohort consisted of 2,280 reperfused STEMI patients.

Age, previous IHD and diabetes, Killip class, creatinine, hemoglobin and troponin on admission, S2B time and LVEF were predictors of 1-year HHF (Table 4).

**Table 4:** Univariable and multivariable regression model for prediction of heart failure hospitalization based on the derivation cohort.

	Heart failure hospitalization		Heart failure hospitalization	
	Odds Ratio (95%CI)	P	Odds Ratio (95%CI)	P
Age (years)		<0.001		0.025
<40	1.00 (reference)		1.00 (reference)	
40–59	1.83 (0.74–4.54)		1.93 (0.67–5.58)	
60–69	3.55 (1.42–8.84)		2.49 (0.84–7.33)	
70–79	4.67 (1.84–11.82)		2.46 (0.80–7.53)	
> = 80	8.40 (3.18–22.15)		4.38 (1.37–14.04)	
Gender		<0.001	Not significant	
Male	1.00 (reference)			
Female	2.28 (1.72–3.02)			
Ethnicity		0.095	NA	
Chinese	1.00 (reference)			
Malay	1.40 (1.05–1.88)			
Indian	1.24 (0.90–1.70)			
Others	0.70 (0.22–2.24)			
History of diabetes		<0.001		<0.001
No	1.00 (reference)		1.00 (reference)	
Yes	3.33 (2.62–4.24)		2.07 (1.54–2.77)	
History of hypertension		<0.001	Not significant	
No	1.00 (reference)			
Yes	2.73 (2.08–3.57)			
History of dyslipidemia		<0.001	Not significant	
No	1.00 (reference)			
Yes	2.02 (1.58–2.59)			
History of IHD		<0.001		<0.001
No	1.00 (reference)		1.00 (reference)	
Yes	3.14 (2.42–4.08)		2.14 (1.56–2.94)	
Current smoker		0.008	Not significant	
No	1.00 (reference)			
Yes	0.72 (0.57–0.92)			
BMI (kg/m^2^)		0.32	NA	
< = 23	1.00 (reference)			
>23	0.88 (0.67–1.14)			
Killip class on admission		<0.001		<0.001
I	1.00 (reference)		1.00 (reference)	
II	4.70 (3.23–6.84)		2.85 (1.84–4.41)	
III	5.66 (3.57–8.96)		1.95 (1.10–3.47)	
IV	5.20 (3.72–7.27)		2.72 (1.81–4.08)	
Cardiac arrest in ambulance/on admission		0.344	NA	
No	1.00 (reference)			
Yes	1.39 (0.70–2.78)			
Anterior STEMI		<0.001	Not significant	
No	1.00 (reference)			
Yes	1.83 (1.43–2.34)			
Creatinine on admission (µmol/L)		<0.001		<0.001
<70	1.00 (reference)		1.00 (reference)	
70–105	0.89 (0.60–1.30)		1.15 (0.73–1.82)	
106–140	1.75 (1.14–2.67)		1.63 (0.98–2.73)	
141–176	3.97 (2.35–6.71)		3.20 (1.71–6.02)	
177–353	6.46 (3.79–11.02)		2.50 (1.24–5.03)	
> = 354	0.37 (0.05–2.74)		0.15 (0.02–1.31)	
Hemoglobin on admission (g/dL)		<0.001		0.003
<10	1.00 (reference)		1.00 (reference)	
10–11	1.25 (0.60–2.60)		1.57 (0.57–4.28)	
12–13	0.52 (0.26–1.04)		0.91 (0.34–2.39)	
14–16	0.28 (0.14–0.56)		0.62 (0.23–1.63)	
> = 17	0.31 (0.15–0.64)		0.93 (0.34–2.56)	
Elevated first troponin T/I on admission		<0.001		0.049
No	1.00 (reference)		1.00 (reference)	
Yes	2.26 (1.77–2.90)		1.35 (1.00–1.81)	
S2B (minutes)		<0.001		0.003
< = 1800	1.00 (reference)		1.00 (reference)	
>18	1.70 (1.31–2.20)		1.57 (1.16–2.12)	
Lowest LVEF during hospitalization (%)		<0.001		<0.001
<40	1.00 (reference)		1.00 (reference)	
40–50	0.17 (0.13–0.23)		0.25 (0.18–0.34)	
>50	0.03 (0.02–0.06)		0.04 (0.02–0.10)	

CI: confidence interval; IHD: ischemic heart disease; BMI: body mass index; STEMI: ST-segment elevation myocardial infarction; S2B: symptom-to-balloon; LVEF: left ventricular ejection fraction; NA: not applicable

Calibration of the predictions from this model was assessed by comparing the average predictions to the actual HHF across deciles and was found to be excellent (Fig. 1). Internal validation showed good fit of the models with a c-statistic of 0.868 (95%CI 0.848–0.888) for 1-year HHF.

External validation showed that the model performed very well with a c-statistic of 0.855 (95%CI 0.823–0.888), with misclassification rate of 24.0% (Fig. 2).

**Figure 3:**
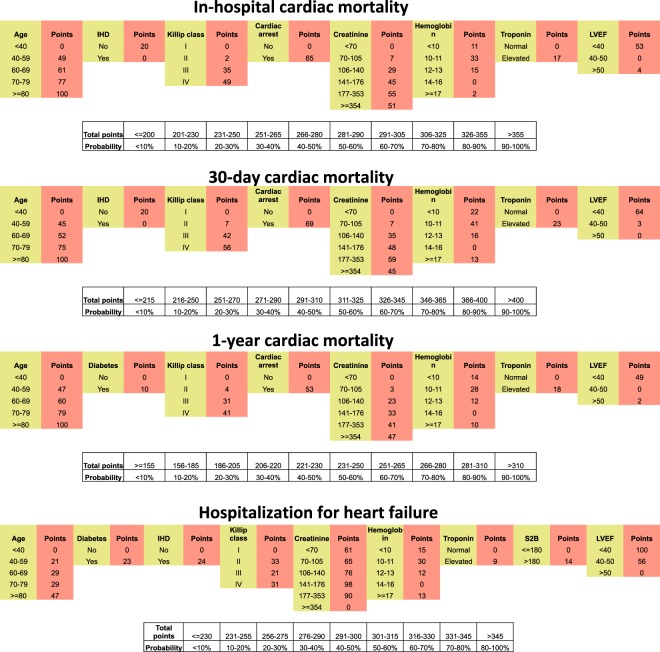
Nomograms to predict cardiac mortality and 1-year HHF for individual patients.

Figure 3 shows the variables that made up each risk score and the score associated with each variable. These risk scores could potentially be incorporated into a calculator to facilitate usage in the clinical setting.

## Discussion

The strength of the SMIR is that it is a mandatory registry and as such, all STEMI patients treated by PPCI between 2008 and 2013 were captured in a cohort of multi-ethnic Asian STEMI patients, comprising predominantly of Chinese, Malays and Indians, reperfused by PPCI in the current era. Unlikely other risk scores predicting all-cause mortality, we have identified independent predictors of cardiac mortality. All these variables are available either at the time of admission or within the first few hours following admission and can be used to predict in-hospital, 30-day and 1-year cardiac mortality and 1-year HHF early during hospitalization.

The potential immediate utility of these predictors is to identify those low risk patients who could be discharged early (e.g. after 48–72 hours). Secondly, it could potentially be used to identify those at high risk and who would benefit from more aggressive up-titration of their prognostic medications (beta-blockers, angiotensin converting enzyme inhibitors, aldosterone antagonists etc.) and more frequent follow-ups. Last but not least, this score could also be used when counseling patients prior to discharge regarding their prognosis.

The initial derivation cohort of the GRACE registry consisted of 13,708 patients recruited between 1999 and 2001 and only a third presented with a STEMI and only 15.2% received reperfusion therapy^6^. Furthermore, they only reported in-hospital and 6 months mortality. Although the recruitment was done in 14 countries, none were from Asia. However, the GRACE2 was subsequently expanded to more countries, including hospitals in Asia^12^. The risk profiles and prognosis for STEMI and NSTEMI are different^13,14^ and therefore developing risk scores specific for STEMI and NSTEMI may provide a more accurate prediction of their prognosis. The TIMI risk score has a dedicated score for STEMI^4^. The derivation cohort in the TIMI STEMI risk score was recruited from >800 hospitals^4^. However, it was not clear how many patients were Asians. Although 15,078 patients were enrolled between 1997 and 1998, they only included patients presenting within 6 hours of symptoms onset and only reported 30-day mortality^4^. The derivation cohort of the Controlled Abciximab and Device Investigation to Lower Late Angioplasty Complications (CADILLAC) score^15^ included 2,082 STEMI patients recruited from 1997 to 1999 into a randomized controlled trial. Patients presenting within 12 hours of symptoms onset were included and those in cardiogenic shock at presentation were excluded and therefore patients in this cohort were not representative of the real world population. The Primary Angioplasty in Myocardial Infarction II (PAMI-II) criteria^16^ (for 6-month mortality) and Zwolle PPCI index^17^ (30-day mortality) can also be used to identify low risk patients for early discharge^2^ but they were both derived from non-Asian cohorts with patients recruited in the 1990s, with the former comprising of 3,252 patients from an RCT and the latter including 1,791 patients from a registry for their derivation cohorts. The Korean Acute Myocardial Infarction Registry (KAMIR)^18^ recently reported a risk score for Asians, derived from a cohort of 14,885 patients but it was not clear how the risk score was derived^18^. There was only one Asian ethnic group represented and they combined both STEMI and NSTEMI patients who either underwent invasive reperfusion strategy or were medically managed. They only reported 1-year mortality and they showed that the KAMIR score performed better than the GRACE score both in the NSTEMI and STEMI, highlighting the need for a risk score that is specific to the local population^18^. Most recently, the Acute Coronary Treatment and Intervention Outcomes Network (ACTION) risk score used to predict in-hospital mortality in ACS was reported^19^. They included a total of 243,440 patients and unlike the SMIR, entry in the former registry was voluntary. Moreover, they consisted predominantly of Caucasians and African Americans and they combined both STEMI and NSTEMI in the model. Although they showed that STEMI had worse outcomes in their model, it is likely that the other components of their score would have carried different weight for STEMI and NSTEMI if they were analyzed separately. The SMIR STEMI risk score is novel as it included all consecutive STEMI patients treated by PPCI as part of a compulsory nationwide registry and it can predict in-hospital, 30-day and 1-year cardiac mortality in a specific group of patients (STEMI patients reperfused by PPCI) in a multi-ethnic Asian cohort. Table 5 summarizes the prognostic variables included in the SMIR STEMI risk score in comparison to variables in the GRACE, TIMI STEMI, CADILLAC, PAMI-II, Zwolle, KAMIR and ACTION scores. All variables were present in at least one of the other risk scores in one form or another.

**Table 5:** Established risk score from GRACE, TIMI STEMI, CADILLAC, PAMI, Zwolle, KAMIR and ACTION.

Outcome	SMIR	GRACE^12^	TIMI-STEMI^4^	CADILLAC^15^	PAMI-II^16^	Zwolle^17^	KAMIR^18^	ACTION^19^
	In-hospital, 30-day and 1 year cardiac mortality and 1-year HHF	6-month all-cause mortality	1-year all-cause mortality	1-year all-cause mortality	6-month all-cause mortality	30-day all-cause mortality	1-year AMI mortality	In-hospital all-cause mortality
c-statistic	0.88–0.92	0.82	0.65	0.83	0.78	0.91	0.83	0.88
Age	Yes	Yes	Yes	Yes	Yes	Yes	Yes	Yes
History of diabetes	Yes		Yes		Yes		Yes (admission glucose)	
History of hypertension	Not significant		Yes					
History of IHD	Yes		Yes (angina)					
BMI/ weight	Not significant		Yes (weight)					
Killip class	Yes	Yes	Yes	Yes	Yes	Yes	Yes	Yes
Cardiac arrest	Yes	Yes						Yes
Anterior STEMI	Not significant	Yes (ST-segment deviation)	Yes (Anterior or LBBB)		Yes (Anterior or LBBB)	Yes		
Creatinine	Yes	Yes		Yes (eGFR <60 ml/min)			Yes	Yes (CrCl)
Hemoglobin	Yes			Yes (Anemia)				
Troponin T/I	Yes	Yes						Yes
Ischemic time	Yes (>3 hours)		Yes (>4 hours)			Yes (>4 hours)		
LVEF during hospitalization	Yes (baseline LVEF <40%)			Yes (baseline LVEF <40%)			Yes (baseline LVEF <40%)	
Heart rate	Not available	Yes	Yes		Yes			Yes
Blood pressure	Not available	Yes	Yes					Yes
3-vessel disease	Not available			Yes		Yes		
Post PPCI TIMI flow	Not available			Yes		Yes		

HHF: hospitalization for heart failure; STEMI: ST-segment elevation myocardial infarction; BMI: body mass index; IHD: ischemic heart disease; LVEF: left ventricular ejection fraction; PPCI: primary percutaneous coronary intervention; TIMI: thrombolysis in myocardial infarction.

### Limitations

We did not perform a direct comparison of the performance of the SMIR STEMI risk score against the GRACE score, which is currently the most popular, due to data on heart rate and blood pressure not being collected on admission. Less than 3% of the reperfused STEMI patients underwent thrombolysis from 2008 to 2015 and were not included in this analysis. Therefore whether the SMIR STEMI risk score would perform well in those Asian countries where thrombolysis is still widely used for STEMI^1^ remains to be investigated. Angiographic data were not available and were not included in the score. Furthermore procedural data (culprit only PCI or multi-vessel PCI) were also not available. However, a previous study^20^ have shown that the angiographic variables provided only a minor improvement in the c-statistic of a risk score to predict in-hospital mortality in patients undergoing PCI procedures. We did not include events occurring during hospitalization that could have improved the risk prediction for 30-day and 1-year cardiac mortality, as we wanted to keep the model simple, for easy adoption in the clinical setting. Only the admission troponin was included in the model as the discharge troponin was performed at variable time point and the assays used were not standardized among the various hospitals. Therefore it was not possible to provide reliable data regarding how the latter correlated with outcomes. The follow-up period was 1 year only and a longer period of follow-up would have provided further valuable information on the longer-term outcomes of these patients. As the risk score for HHF excluded patients who died due to non-HF causes within 1 year from STEMI discharge and without HF admission, the competing risk from non-HF death may have biased the HHF risk score and undermined its usefulness. Last but not least, we used the ICD codes to identify HHF endpoints and the latter has been shown to fail to capture all HHF events in western health care system^21^. Whether this holds true in the Singapore health care system with a dedicated team to collect all outcomes in this compulsory nationwide registry remains to be seen.

## Conclusion

We used a real-world, national AMI registry to identify independent predictors of in-hospital, 30-day and 1-year cardiac mortality and 1-year HHF in a multi-ethnic Asian STEMI patients reperfused by PPCI. We have provided the variables and their associated scores that could be incorporated into a risk score to risk-stratify patients and guide duration of hospital stay, short and medium term management and follow-up, to improve outcomes in these patients.

## Methods

### Population

This was a retrospective study on data collected prospectively between 2008 and 2015 by the National Registry of Diseases Office in SMIR. Data collection on all AMI cases from all public and private hospitals is mandated and funded by the state and yearly reports are generated for the Ministry of Health^11,22,23^. Ethics approval was obtained from the SingHealth Centralized Institutional Review Board and the requirement for patient consent was waived. The study was conducted according to the Declaration of Helsinki.

### Inclusion and Exclusion criteria

**Figure 4:**
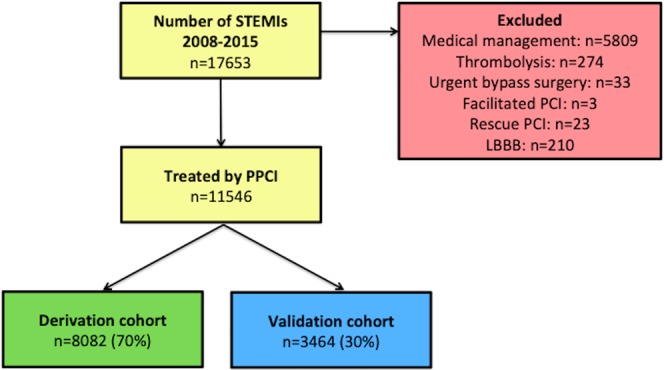
Flow chart of STEMI patients from the SMIR included in this study.

The main inclusion criteria were patients presenting to hospital with a STEMI within 12 hours of symptoms onset and were reperfused by PPCI. Patients with a STEMI but not reperfused by PPCI or those with a LBBB were excluded as shown in the flow chart in Fig. 4.

### Clinical outcomes of interest

The main outcomes of interest were in-hospital cardiac mortality, 30-day cardiac mortality, 1-year cardiac mortality and 1-year HHF. Specifically for HHF, the data was available for 2008 to 2013 only. Patients who died during hospitalization for STEMI were excluded for this outcome. Among patients discharged alive, patients who died due to HF within 1 year from STEMI discharge but without HF admission were included. However, patients who died due to non-HF causes within 1 year from STEMI discharge and without HF admission were excluded.

### Data collection

Registry coordinators confirmed all diagnosis of STEMI from physical and electronic medical records and data on patients’ demographics, comorbidities, location of STEMI on ECG, initial blood results such as hemoglobin, creatinine and troponin T or I were extracted. Details on Killip class and cardiac arrest in the ambulance or on admission (prior to transfer to the cardiac catheterization laboratory) and S2B/ D2B times were also recorded. The International Classification of Diseases 9th Revision (ICD-9 Clinical Modification) code 410 was used to identify STEMI cases diagnosed from 2008 to 2011, whereas ICD-10 codes I21 and I22 were used for STEMI cases diagnosed from 2012 to 2015. Data on admission heart rate and blood pressure were not available in this registry.

STEMI was defined by a typical chest pain of 20 minutes and significant ST-segment elevation (0.1 or 0.2 mV on two adjacent limb or precordial leads), and with a corresponding rise in cardiac biomarkers. Troponin T or I performed on admission was defined as abnormal if they were greater than the 99^th^ percentile of the reference range from each hospital laboratory.

Cardiac mortality was identified as death occurring from a cardiac cause and defined by the ICD codes (ICD-9: 391–398, 402, 410–429; ICD-10: I00-I52 except I26). The time of death was extracted from death certificates obtained from the Registry of Births and Deaths. As the reporting of death is mandatory for all Singapore residents and the vital statuses of study population (patients with STEMI in January 2008 to December 2015) were matched till 30 June 2017, a date that is beyond one year from the last STEMI case included in this study, no patient was lost to follow-up for all mortality outcomes.

HHF was identified using ICD codes for heart failure admission (ICD-9: 412, 414.10, 414.19, 414.8, 428, 428.20, 428.21, 428.22, 428.23, 428.30, 428.31, 428.32, 428.33, 428.40, 428.41, 428.42, 428.43, 428.0, 428.9; ICD-10: I25.2, I25.3, I25.5, I50.1, I50.20, I50.21, I50.22, I50.23, I50.30, I50.31, I50.32, I50.33, I50.40, I50.41, I50.42, I50.43, I50.9). No patient was lost to follow-up for HHF as all admitted patients will have a discharge diagnosis. However, as the admission data was available till 31 December 2014, 1-year HHF could be matched for patients with STEMI in 2008 to 2013 only.

### Statistical analysis

Continuous variables were expressed as medians with interquartile ranges and categorical variables were expressed as percentages. All analysis was performed using StataCorp. 2013. *Stata Statistical Software: Release 13* (College Station, TX: StataCorp LP). The statistical methodology was similar to that used for the GRACE score derivation^6^. Random selection was performed to divide the cohort into two groups with 70% of the cohort included in the derivation cohort and 30% in the validation cohort. Variables that were prognostic from the GRACE, TIMI STEMI and CADILLAC^15^ risk scores and that were available from the SMIR were included in the univariable logistic regression. Other potentially prognostic variables such as cardiovascular risk variables, ethnicity, gender, S2B and D2B were also included in the univariable logistic regression model. Odd ratios with 95% confidence interval (95%CI) were used to assess the relationship between the included variables and cardiac mortality during hospitalization, within 30 days and within 1 year and 1-year HHF.

Multivariable stepwise logistic regression with backward elimination was subsequently used to determine the significant predictors of cardiac mortality and 1-year HHF among all the variables included in the univariable logistic regression. Variables that were significant with a P value of <0.05 were included in the final multivariable logistic models. Missing data were handled as case deletion without any imputation. Internal validity was assessed using bootstrapping techniques on the derivation cohort. External validity was assessed using the validation cohort. C-statistic (area under the curve) with 95%CI derived from Hosmer-Lemeshow test was used to assess the goodness of fit of the final multivariable logistic models. A model with a c-statistic of >0.800 was considered to have good fit. Misclassification rate derived from linear discriminant analysis was used to assess the discriminatory power of the final multivariable logistic models. The final multivariable logistic models were used to develop nomograms for patient risk^6^.
